# How does nonsyndromic craniosynostosis affect on bone width of nasal cavity in children? – Computed tomography study

**DOI:** 10.1371/journal.pone.0200282

**Published:** 2018-07-13

**Authors:** Katarzyna Gruszczyńska, Wirginia Likus, Magdalena Onyszczuk, Rita Wawruszczak, Kamila Gołdyn, Zbigniew Olczak, Magdalena Machnikowska-Sokołowska, Marek Mandera, Jan Baron

**Affiliations:** 1 Department of Diagnostic Imaging, School of Medicine in Katowice, Medical University of Silesia, Katowice, Poland; 2 Department of Anatomy, School of Health Sciences in Katowice, Medical University of Silesia, Katowice, Poland; 3 Regional Specialist Hospital in Tychy, Megrez Sp. z o.o., Tychy, Poland; 4 Students’ Scientific Organization, Department of Radiology and Nuclear Medicine, School of Medicine in Katowice, Medical University of Silesia, Katowice, Poland; 5 Department of Diagnostic Imaging and Intervention Radiology, The Independent Public Clinical Hospital no. 6 of the Medical University of Silesia in Katowice, John Paul II Upper Silesian Child Health Centre, Katowice, Poland; 6 Department of Emergency Medicine and Pediatric Neurosurgery, School of Public Health in Bytom, Medical University of Silesia, Katowice, Poland; 7 Department of Radiodiagnostics and Invasive Radiology, School of Medicine in Katowice, Medical University of Silesia, Katowice, Poland; Ohio State University, UNITED STATES

## Abstract

Craniosynostosis is caused by premature fusion of one or more cranial sutures, restricting skull, brain and face growth. Nonsyndromic craniosynostosis could disturb the proportions of face. Although morphometric diameters of nasal cavity in healthy children are already known, they have not been established yet in children with nonsyndromic craniosynostosis. The aim our study was to check whether diameters of bone structures of nasal cavity in children with nonsyndromic craniosynostosis measured in CT are within normal range. 249 children aged 0–36 months (96 with clinical diagnosis of nonsyndromic craniosynostosis and 153 in control group) were included into the study. The following diameters were measured on head CT scans: anterior bony width (ABW), bony choanal aperture width (BCAW), right and left posterior bony width (between bone sidewall and nasal cavity septum—RPBW and LPBW). The study group has been divided into 4 categories, depending on child’s age. The dimensions measured between bone structures of nasal cavity were statistically significantly lower in comparison to the control group. They did not depend on the sex for ABW, nor on age in groups 7–12 months and < 2 years for BCAW, RPBW and LPBW. The measured dimensions increased with age. In children with nonsyndromic craniosynostosis the diameter of pyriform aperture and bony choanal aperture were lower than in controls, what may be described as fronto-orbital anomalies. Morphometric measurements of anthropometric indicators on CT scans could be used as standards in the clinical identification of craniosynostosis type and may help in planning surgical procedures, particularly in the facial skeleton in children.

## Introduction

Craniosynostosis, a pathologic fusion of calvarial bones that is associated with an abnormal skull growth, was first described in 1830 by Otto [1]. It is a congenital disorder, most often (60–80%) presenting itself either as a nonsyndromic (isolated) feature, or as a part of Apert, Crouzon or Pfeiffer syndrome. Craniosynostosis occurs in 1 in 2500 births [2]. Nonsyndromic subtype is present in 0.4 to 1 infants per 1000 births [3]. Craniosynostosis is controlled by several genetic mutations involving transcription factor, growth factor receptor and cytokine expression. These numerous factors are associated with premature suture fusion [4–7]. In children, nonsyndromic craniosynostosis is more commonly encountered than syndromic cases in craniofacial surgery. Although the abnormal shape of the calvarium is the main symptom of the disease, the development of the face is also disturbed [8–11]. In children with syndromic craniosynostosis obstruction of the rhinopharynx leading to sleep apnea can occur [12–14]. In children with nonsyndromic craniosynostosis, the disturbed proportion of the face, with hypotelorism, and diminished interorbital diameter is often observed. However, despite numerous anthropometric and CT-based measurements of the skull, including the most popular cephalic index, the data pertaining to the morphometric measurements of the nasal cavity in children with nonsyndromic craniosynostosis are limited [15–17]. Potentially, the developmental restriction of nasal cavity in these children could have clinical implications, as breathing through the nose is the only way available to neonates and small children. In our previous study we established, on the basis of CT, the normal values of nasal cavity measurements in healthy children aged 0–3 years [18]. The goal of the present study was to check whether the diameters of bone structures of nasal cavity in children with nonsyndromic craniosynostosis measured in CT are within this normal range. Because the first 2 years of life are a period of a very intense growth of the organism and substantial percentage increase of body length, the authors divided the study group into 4 narrow age ranges, to check if there were statistically significant differences between the parameters analyzed in adjacent age groups and if they could be analyzed together. The second reason for division of both children groups (controls and those suffering from nonsyndromic craniosynostosis) into four narrow age categories was to perform a more precise comparison of studied morphological parameters reducing the potential influence of aging and growth.

## Materials and methods

### Patients

**Fig 1:**
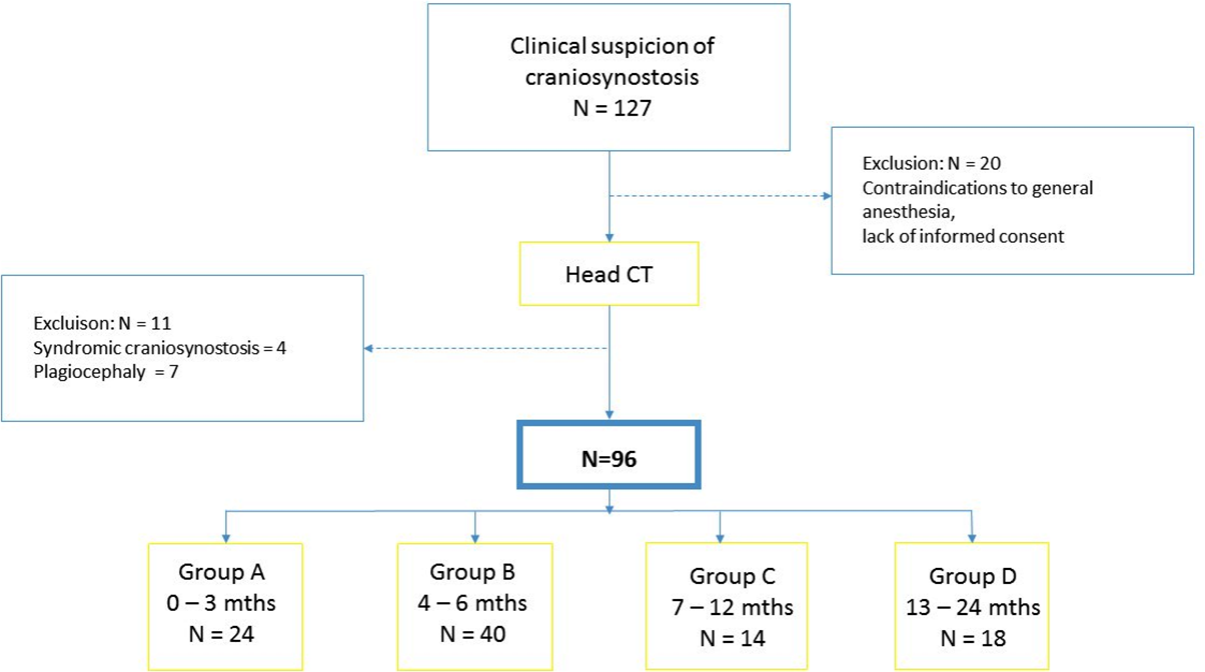
Study material. Exclusion criteria and examined groups divided according to age.

A retrospective study was performed on 127 Caucasian children who underwent head CT due to clinical suspicion of craniosynostosis in the Upper Silesian Children`s Health Center in Katowice. The inclusion criterion was craniosynostosis diagnosed on CT scan. 96 patients (28 girls and 68 boys), aged 0–24 months were included in the study. Children with contraindications to general anesthesia, in which ‘feed and wrap’ technique had been working, or without written parents’ permission were excluded from the study. In 7 children, no suture fusion was found on CT (false plagiocephaly), and in further 4 children genetic syndrome was recognized (single cases of Apert and Crouzon syndromes and two cases of Noonan syndrome). These patients were also excluded from the group. (Fig 1).

The study group has been divided into 4 age categories (Table 1). Permission of Commission of Bioethics of the Medical University of Silesia in Katowice was received, and parents of all participating children consented for CT scans, as well as for the intravenous injection of ionized contrast media. The control group consisted of 153 Caucasian children (74 females, 79 males), without any craniofacial abnormalities, who underwent CT because of trauma (without skull fracture on CT). The children were divided into four age groups (0–3, 4–6, 7–12 and 12–24 months), the same as in children with craniosynostosis (Table 1).

**Table 1:** Children with craniosynostosis and from the control group, divided according to age.

Age group	Age categories	Female (n)		Male (n)		Total	
		craniosynostosis	control	craniosynostosis	control	craniosynostosis	control
**A**	0–3 mths	6	13	18	10	24	23
**B**	4–6 mths	9	20	31	15	40	35
**C**	7–12 mths	8	27	10	26	18	53
**D**	13–24 mths	5	14	9	28	14	42
	**Total**	**28**	74	**68**	79	**96**	153

Analyzing the distribution of particular types of craniosynostosis in the study group, the most numerous group were children with sagittal synostosis (53.1%), then with metopic synostosis (30.2%). Coronal synostosis was reported in 9.4% of children. The least numerous was group with combined craniosynostosis and unilateral lambdoid synostosis (5.2% and 2.1% respectively). For the detailed description, see S1 Table.

### CT examination

CT examinations were performed with a CT scanner Aquilion S (Toshiba, Tokyo, Japan). The standard vendor diagnostic protocols for head CT were modified according to patients’ age, with the following parameters: spiral mode, 80–100kV tube voltage, 250mAs tube current, gantry rotation time 0.75s, FOV 240. All head from *gonion* to *vertex*, was included into the field of view, together with soft tissue of the face. The scanner gantry was not tilted. The reconstructed slice thickness was 0.5mm, with increments of 0.5mm. Only one acquisition occurred after i.v. injection of contrast (volume 1ml per 1kg of body weight, Iopromidum, 30g Iodine/100ml, Bayer).

### Nasal cavity measurements

The measurements were performed twice by one operator on the workstation (Dicom viewer OsiriX 64-bit by Alteris), on axial scans and 2D multiplanar reformations (2D MPR) parallel to the palatine bone at the level of nasal cavity floor. All measurements were performed directly on CT films; each measurement was taken with the accuracy of 1/100 mm; measurements were standardized to a 5 cm reference scale on each film. The average of the two separate measurements was used for analysis.

**Fig 2:**
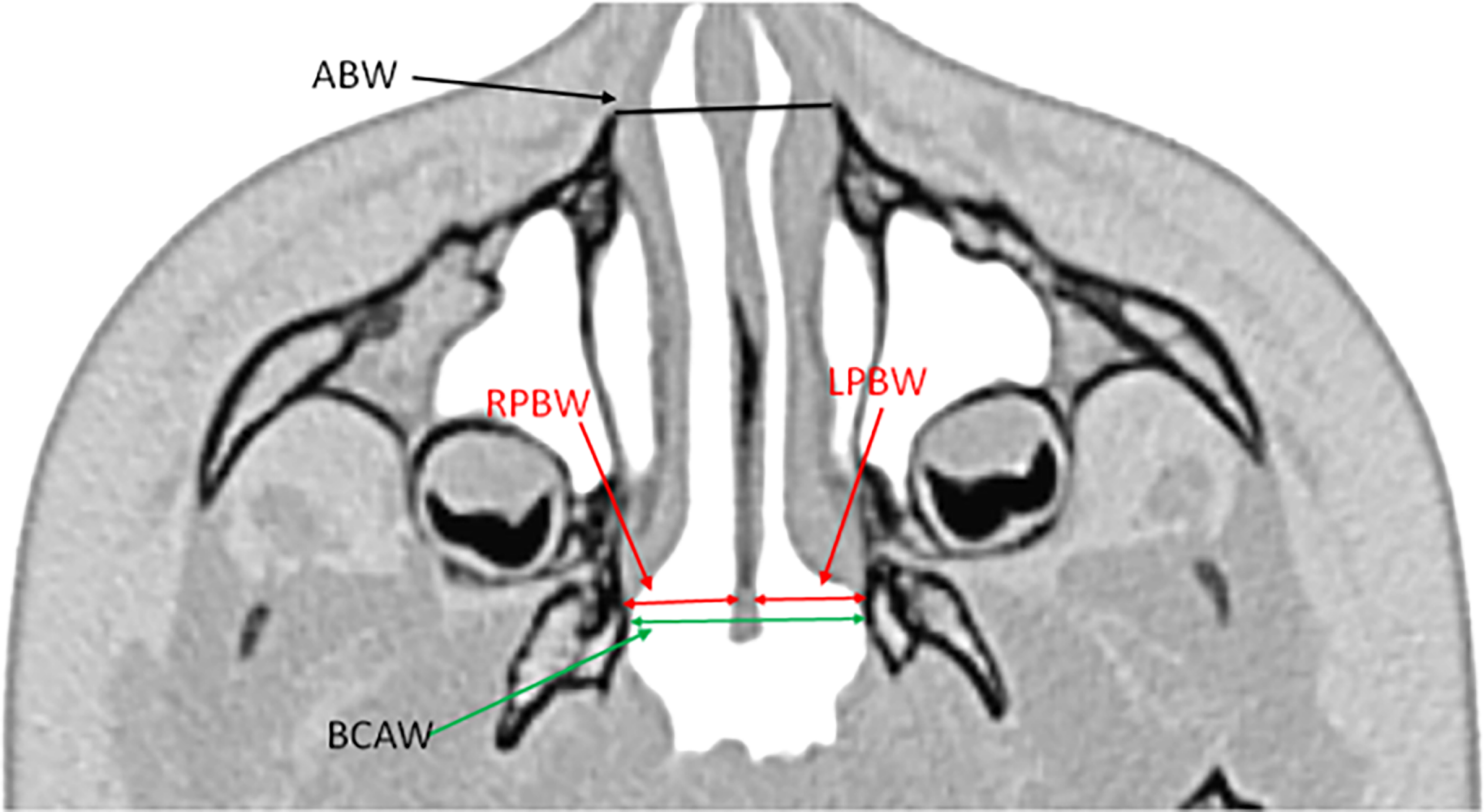
Measurements of nasal cavity dimensions in children with craniosynostosis on CT image, with colour inversion. Abbreviations: ABW—anterior bony width; BCAW—bony choanal aperture width; RPBW—right posterior bony width; LPBW—left posterior bony width.

The following dimensions of the nasal cavity were measured (Fig 2):

ABW—anterior bony width between the two ridges extruding from the maxilla-pyriform aperture,
BCAW—bony choanal aperture width between both pterygoid processes—choanal aperture,
RPBW—right posterior bony width between bone sidewall and septal mucosa,
LPBW—left posterior bony width between bone sidewall and septal mucosa.

### Statistical analysis

Shapiro-Wilk test was used to check the consistence of empirical distribution of evaluated variables with normal distribution. Levene test was applied to assess the homogeneity of variance. The one-way analysis of variance (ANOVA) was used for the assessment of differences between the age groups. To evaluate the significance of differences between the sexes in each age group the Cochran Cox test was used. The measured dimensions were compared with the previously established measurements in the control group of 153 children (Cochran Cox test). The statistical difference between groups has been assessed at the level of p≤0.05.

The “Reduction” of the measured parameter between patients and controls (last column) was calculated as follows: the percentage of reduction is the difference between the average dimension in healthy children and the average dimension in children with craniosynostosis, expressed as the percentage of the average value in healthy children.

The following software was used for the calculation and visualization of results: Statistica 12.0 StatSoft® and MS Excel 2013.

## Results

After application of strict exclusion criteria 96 CT scans (28 from females and 68 from males) have been subjected to the analysis. The detailed results of all measurements (mean±SD, 95% CI, minimum, maximum) have been listed in Tables 2–5.

**Table 2:** Anterior bony width (ABW) of nasal cavity in children with craniosynostosis according to sex and age.

Patients' group		ABW–anterior bony width [mm]					*p* (M vs F)	*Reduction compared to control group [mm]*
		Mean	SD	95% CI	min.	max.		
**A**	All	12.13	2.89	10.91–13.35	7.08	17.76		2.74±1.27 18.5%
	M	12.26	2.80	10.86–13.65	9.25	17.76	ns	
	F	11.76	3.37	8.22–15.29	7.08	17.44		
**B**	All	12.20	2.65	11.35–13.05	7.2	19.33		3.40±1.50 21.8%
	M	11.94	2.55	11.01–12. 88	7.2	17.98	ns	
	F	13.08	2.95	10.82–15.35	9.72	19.33		
**C**	All	13.72	2.21	12.63–14.82	9.42	16.97		3.39±0.93 19.8%
	M	13.84	2.22	12.25–15.43	10.66	16.97	ns	
	F	13.57	2.33	11.62–15.53	9.42	16.57		
**D**	All	15.32	3.46	13.32–17.31	10.11	19.52		3.15±1.15 17.0%
	M	15.15	3.56	12.42–17.89	10.11	19.52	ns	
	F	15.61	3.65	11.41–20.15	10.63	18.9		

A (0–3 mths); B (4–6 mths); C (7–12 mths); D (13–24 mths); F: female, M: male, SD: standard deviation, 95% confidence intervals, min—minimum, max—maximum; ns–not significant.

**Table 3:** Choanal aperture (BCAW) of nasal cavity in children with craniosynostosis according to sex and age.

Patients' group		BCAW–bony choanal aperture width [mm]					*p* (M vs F)	*Reduction compared to control group [mm]*
		Mean	SD	95% CI	min.	max.		
**A**	All	13.53	1.71	12.86 - 14.26	10.63	16.22		3.27±0.29 17.0%
	M	13.92	1.42	13.21 - 14.63	10.63	16.22	***p*<0.00001**	
	F	12.38	2.10	10.18–14.58	10.89	15.54		
**B**	All	14.67	1.44	14.21–15.14	11.8	18.99		2.00±0.18 12.2%
	M	15.17	1.19	14.73–15.61	11.95	18.99	***p*<0.00001**	
	F	12.97	0.79	12.36–13.57	11.8	14.4		
**C**	All	15.36	2.42	14.15–16.56	8.57	18.81		2.41±0.96 13.5%
	M	15.53	1.71	14.31–16.75	12.54	17.50	ns	
	F	15.14	3.22	12.45–17.84	8.57	18.81		
**D**	All	16.65	2.19	15.39–17.92	12.57	20.23		2.6200B10.72 16.9%
	M	16.74	1.70	15.38–18.10	14.68	20.23	ns	
	F	16.50	3.04	12.72–20.28	12.57	19.17		

A (0–3 mths); B (4–6 mths); C (7–12 mths); D (13–24 mths); F: female, M: male, SD: standard deviation, 95% confidence intervals, min—minimum, max—maximum; ns–not significant.

**Table 4:** Right posterior bony width (RPBW) of nasal cavity in children with craniosynostosis according to sex and age.

Patients' group		RPBW—right posterior bony width [mm]					*p* (M vs F)	*Reduction compared to control group [mm]*
		Mean	SD	95% CI	min.	max.		
**A**	All	6.06	1.28	5.52 - 6.60	2.91	7.71		0.52±0.12 8.5%
	M	6.45	0.82	6.08 - 6.92	4.1	7.71	***p* = 0.036**	
	F	4.75	1.53	3.14–6.36	2.91	6.96		
**B**	All	6.74	0.93	6.45–7.04	4.21	8.23		0.18±0.12 2.6%
	M	6.98	0.76	6.70–7.26	4.98	8.23	***p* = 0.025**	
	F	5.93	1.05	5.12–6.73	4.21	7.4		
**C**	All	6.94	1.24	6.32–7.55	4.21	8.58		0.71±0.35 9.3%
	M	6.77	1.19	5.91–7.62	4.7	8.2	ns	
	F	7.15	1.35	6.02–8.28	4.21	8.58		
**D**	All	7.77	1.04	7.17–8.37	6.32	9.82		0.59±0.41 7%
	M	7.67	0.92	6.95–8.38	6.32	9.24	ns	
	F	7.96	1.32	6.32–9.60	6.44	9.82		

A (0–3 mths); B (4–6 mths); C (7–12 mths); D (13–24 mths); F: female, M: male, SD: standard deviation, 95% confidence intervals; min—minimum, max—maximum; ns–not significant.

**Table 5:** Left posterior bony width (LPBW) in children with craniosynostosis according to sex and age.

Patients' group		LPBW–left posterior bony width [mm]					*p* (M vs F)	*Reduction compared* *to control group* *[mm]*
		Mean	SD	95% CI	min.	max.		
**A**	All	6.06	6.06	5.64 - 6.48	3.42	7.44		0.50±0.13 8.5%
	M	6.28	6.28	5.99 - 6.56	5.36	7.25	ns	
	F	5.40	5.40	3.68–7.12	3.42	7.44		
**B**	All	6.64	6.64	6.30–6.98	4.23	8.24		2.81±0.25 4.1%
	M	6.95	6.95	6.64–7.27	4.46	8.24	***p = 0*.*025***	
	F	5.55	5.55	4.79–6.32	4.23	0.70		
**C**	All	6.71	6.71	6.18–7.41	4.36	8.40		0.94±0.32 12.3%
	M	6.92	6.92	6.00–7.84	4.75	8.40	ns	
	F	6.64	6.64	5.62–7.67	4.36	8.13		
**D**	All	7.55	7.55	6.87–8.23	5.87	9.45		0.81±0.55 9.7%
	M	7.39	7.39	6.75–8.03	5.91	8.85	ns	
	F	7.83	7.83	5.68–9.57	5.87	9.45		

(0–3 mths); B (4–6 mths); C (7–12 mths); D (13–24 mths); F: female, M: male, SD: standard deviation, 95% confidence intervals, min—minimum, max—maximum; ns–not significant.

In all age groups of children with craniosynostosis, each of the analyzed dimensions of the nasal cavity increased with the age of a child. There were no statistically significant differences between the boys and the girls for anterior bony width (ABW) in all analyzed age groups (Table 2). A similar statistically insignificant difference between boys and girls was reported in the children in the reference group for the ABW parameter. The width of the bony choanal aperture width (BCAW) in the group of children aged 0–3 months and 4–6 months was statistically significantly higher in girls than in boys in the same age groups. No differences have been noted between the sexes in the remaining age groups (Table 3). For children in the reference group (healthy children), the BCAW dimension was significantly higher in boys than in girls in the 3–6 months age group. The similar pattern of growth was observed for the right posterior bone width (RPBW). There were no statistically significant differences between the sexes, except for the oldest groups of children, aged 7–12 months and 13–24 months (Table 4). Comparing children from the reference group, only in the youngest infants (0–3 months) this dimension was significantly higher in boys compared to girls. In the case of left posterior bony width between bone sidewall and septal mucosa (LPBW), statistically significant difference between the sexes was observed only in children aged 4–6 months (Table 5). In control group there were no statistically significant differences in LPBW dimension between sexes.

Comparison of the differences between the mean values of the measured dimensions of nasal cavity in children, regardless the sex, between all four age groups (Table 6), revealed that there were no differences in the measured dimensions between the youngest children from groups A and B, both for children with craniosynostosis and control group as well as the oldest children from groups C and D with craniosynostosis. Additionally, in the group of children with craniosynostosis, there were no statistically significant differences in the measured diameters of the nasal cavity between children from adjacent age ranges. For the LPBW and RPBW dimensions in the craniosynostosis group, statistically significant differences were recorded only between the youngest and the oldest children (Table 6).

**Table 6:** Comparison of nasal dimensions in children with craniosynostosis and reference group of healthy children.

REFERENCE VALUES					CRANIOSYNOSTOSIS			
	B	C	D	**ABW**		B	C	D
A	ns	***	***		A	ns	ns	***
B		***	***		B		ns	***
C			***		C			ns
	B	C	D	**BCAW**		B	C	D
A	ns	ns	***		A	ns	*	***
B		*	***		B		ns	*
C			***		C			ns
	B	C	D	**RPBW**		B	C	D
A	ns	*	***		A	ns	ns	***
B		ns	***		B		ns	ns
C			***		C			ns
	B	C	D	**LPBW**		B	C	D
A	ns	***	***		A	ns	ns	**
B		***	***		B		ns	ns
C			***		C			ns

A (0–3 mths); B (4–6 mths); C (7–12 mths); D (13–24 mths); ns–not significant

*—p≤0.05

**—p<0.001

***—p<0.0001.

**Fig 3:**
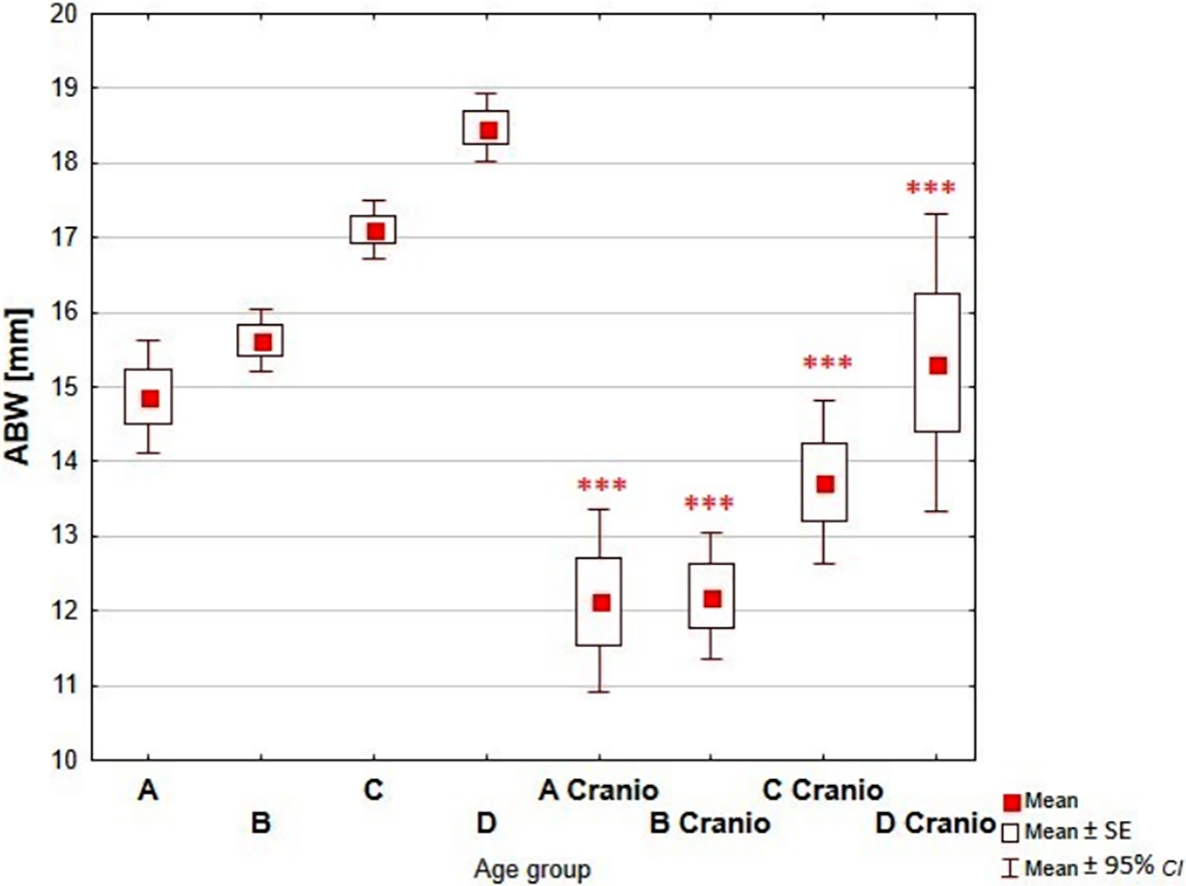
Reference values vs craniosynostosis–ABW. (A-D–reference age groups: A (0–3 mths); B (4–6 mths); C (7–12 mths); D (13–24 mths); A Cranio—D Cranio–children with craniosynostosis age groups; ***—p<0.0001).

**Fig 4:**
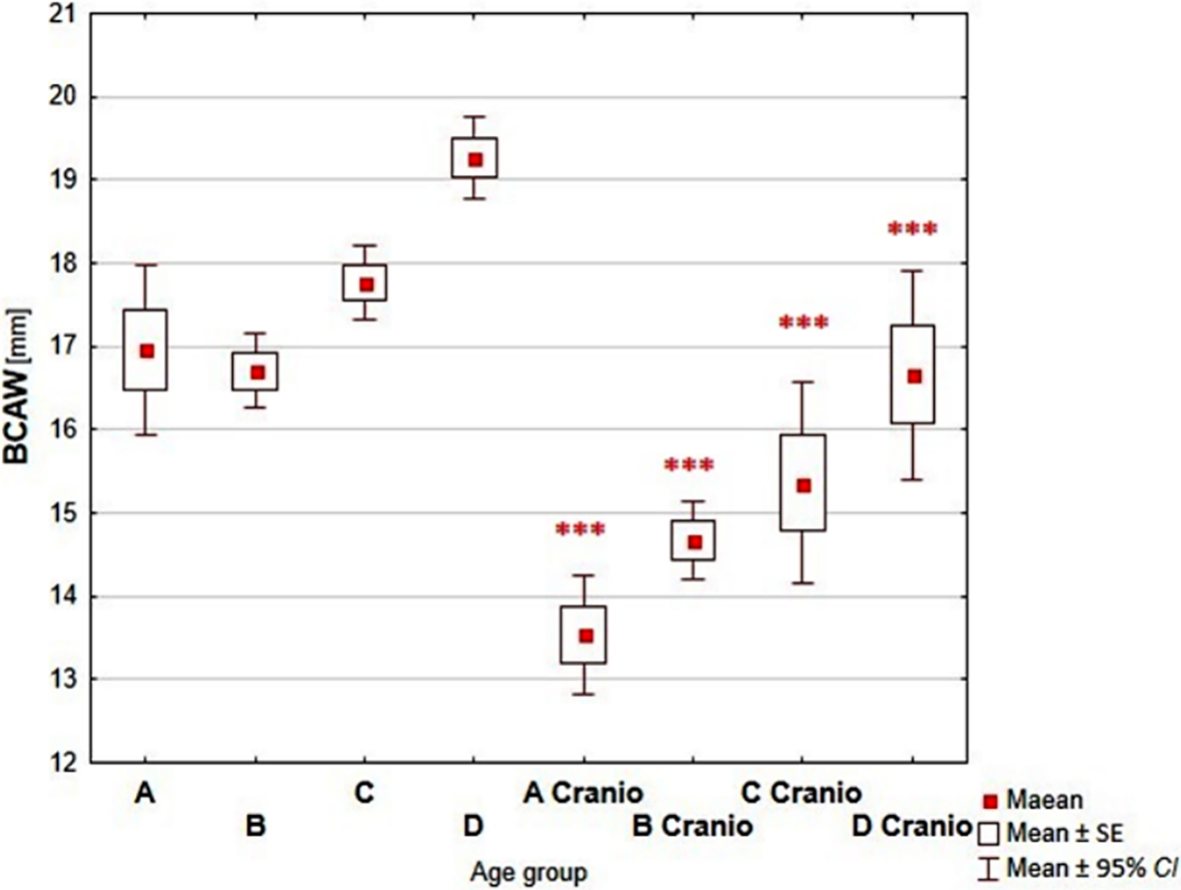
Reference values vs craniosynostosis–BCAW. (A-D–reference groups: A (0–3 mths); B (4–6 mths); C (7–12 mths); D (13–24 mths); A Cranio—D Cranio–children with craniosynostosis age groups; ***—p<0.0001).

**Fig 5:**
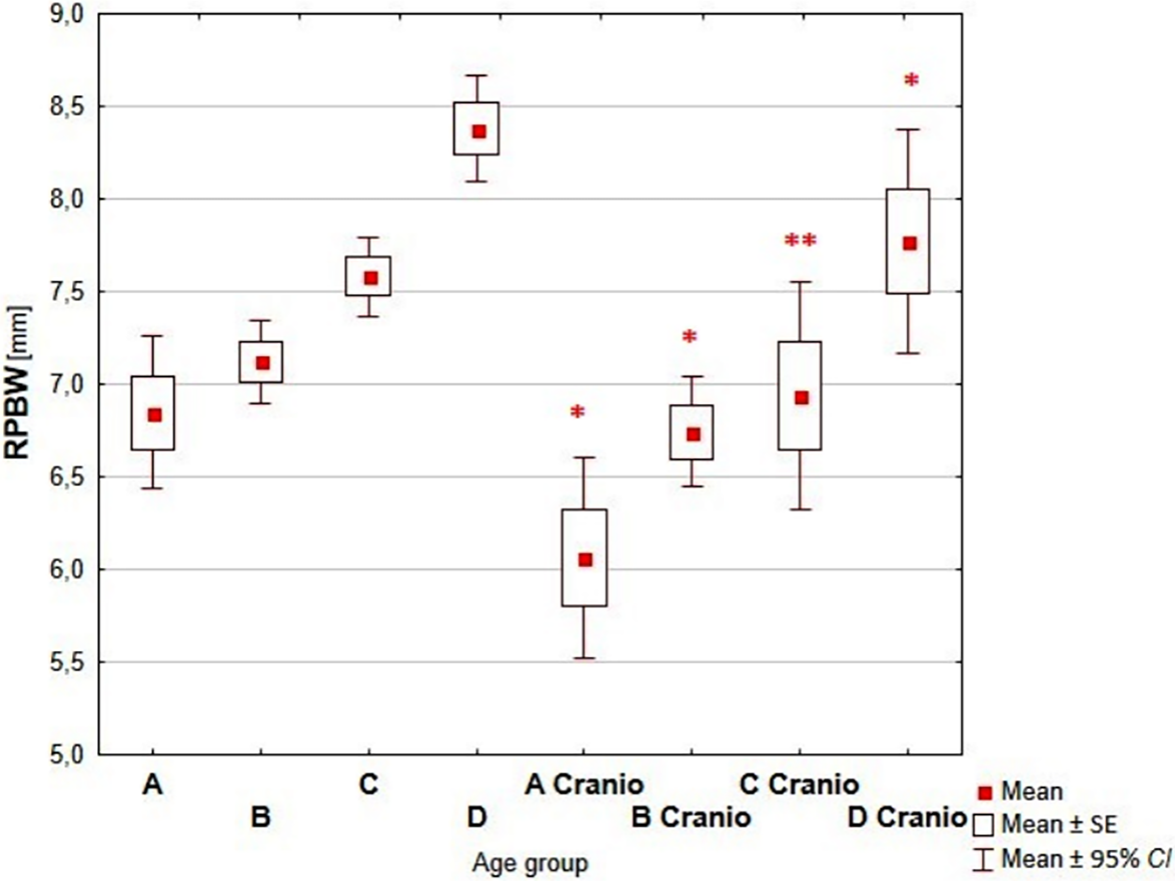
Reference values vs craniosynostosis–RPBW. (A-D–reference age groups: A (0–3 mths); B (4–6 mths); C (7–12 mths); D (13–24 mths); A Cranio—D Cranio–children with craniosynostosis age groups; *—p<0.05; **—p<0.001).

**Fig 6:**
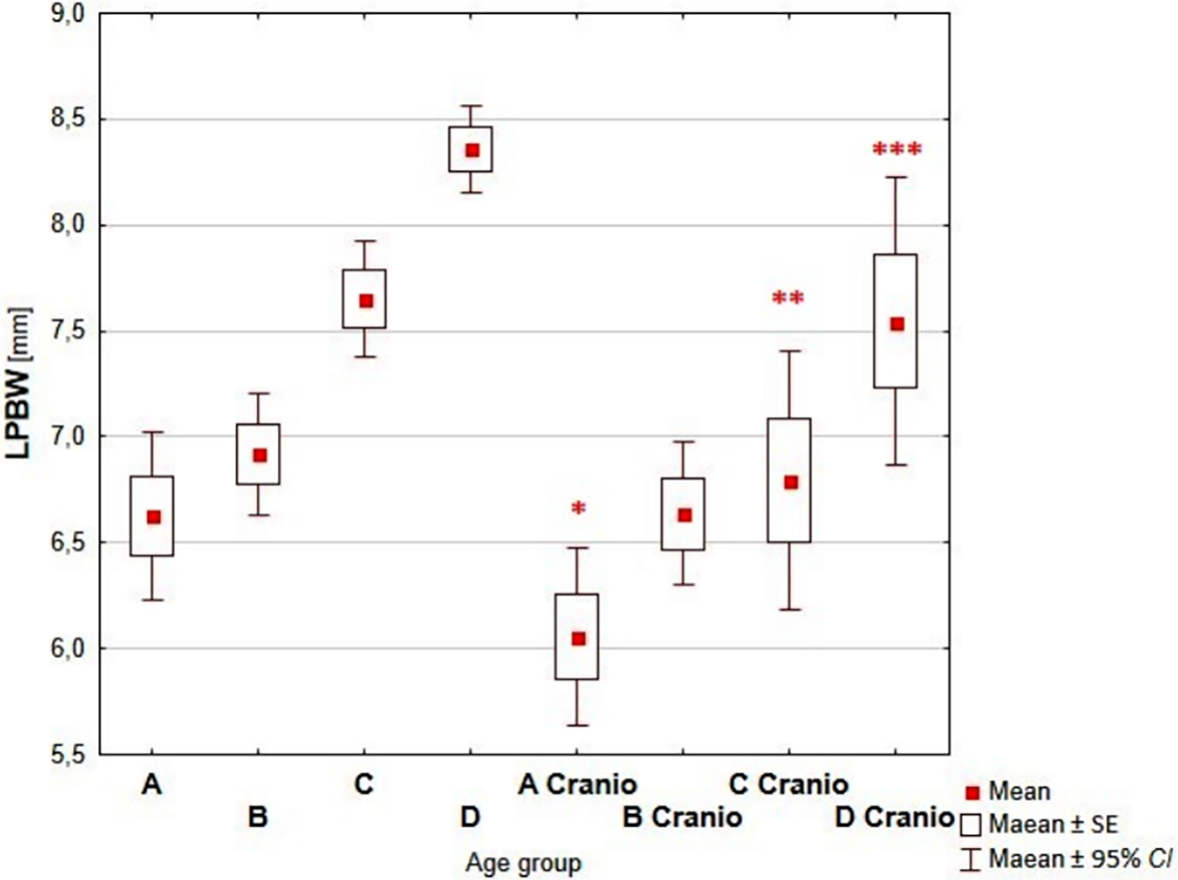
Reference values vs craniosynostosis–LPBW. (A-D–reference age groups: A (0–3 mths); B (4–6 mths); C (7–12 mths); D (13–24 mths); A Cranio—D Cranio–children with craniosynostosis age groups; *—p<0.05; **—p<0.001; ***—p<0.0001).

Comparative analysis of the dimensions of the nasal cavity, using as reference the group of children without growth abnormalities of the skull, showed a statistically significant increase of measured dimensions with the age of the child in each analyzed age groups (Figs 3–6, Tables 2–5).

The width of the pyriform aperture (ABW dimension) was approximately 20% lower in comparison with the control group. Decrease of the dimension of bony choanal aperture was biggest in groups of the youngest and the oldest children (approximately 17%, compared with reference group).

Analyzing the dimensions of right and left posterior bony width (RPBW and LPBW) the smallest differences between healthy children and children with craniosynostosis were reported in children aged 3–6 months (2.6% and 4%, respectively, Tables 4 and 5) and the most profound differences were observed in children aged 7–12 months (9.3% and 12.3%, respectively; Tables 4 and 5).

## Discussion

Craniosynostosis or premature atresia of cranial sutures is a developmental disorder classified among the so-called bony face deformations [19]. It may occur as an isolated form (nonsyndromic) or as a part of congenital craniofacial syndromes [9,11,20,21]. The aim of our study was to analyze the diameters of nasal cavity in children with isolated (nonsyndromic) craniosynostosis. As the patients with syndromic craniosynostosis (SCS) often suffer from severe underdevelopment of the midface, except the premature fusion of the calvarial sutures, it is necessary to precisely determine the growth pattern not only of the skull, but also of the midface and nasal cavity, before the surgical correction. We would like to emphasize the fact that, in our work we measured for the first time the nasal cavity in children aged 0–2 years with nonsyndromic craniosynostosis. Up to now the diameters of nasal cavity have been measured in neonates suffering from choanal stenosis, as well as in healthy children in different age groups.

In scientific papers the evaluation of the skull of infants and young children with premature atresia of cranial sutures typically refers to the cranial index (CI), brain volume, and the measurements of the jaw or of the skull base [15,17,22–25]. The considerable majority of the articles evaluate these measurements in children with syndromic craniosynostosis. Sometimes, in such children, the altered growth pattern of the skull provides the necessary space for the growing brain, but results in abnormal head shape and abnormal facial features [11,12]. Abnormalities of facial skeleton can cause many functional disorders, such as exophthalmos (protrusion of the eyeball) due to the flat, widely spaced orbits. Also, the reduced volume of the upper respiratory tract, caused by facial and/or jaw hypoplasia, can result in sleep disturbances with following cerebral hypoperfusion [12]. Only a handful of papers report the results of complex measurements of facial skeleton in children with craniosynostosis, particularly comparing the dimensions with those in healthy children [26]. The main aim of such a comparison is to indicate the reference values, which in further perspective could be useful during the diagnosis of a specific type of craniosynostosis and planning surgical procedure.

The purpose of our study was to verify whether the dimensions of upper part of the bony face vary between groups of healthy children and children with craniosynostosis, aged 0–2 years. As a research method, we have chosen CT scans with MPR reconstruction, because the measurements on CT scans are highly reliable [27,28], particularly as we performed our measurements on scans with very low slice thickness (only 0.5 mm).

Based on the obtained results we conclude, that during the growth of the child’s head dimensions of the nasal cavity increase with age, for both the pyriform aperture and choanal aperture. The observed differences in growth rates for both apertures are statistically smaller in comparison to children without premature fusion of cranial sutures. This is confirmed by other studies concerning skull measurements in children. Kowalewska et al. [26] analyzed the shape of the skull and the facial skeleton in children up to 1 year of age with nonsyndromic craniosynostosis. Discriminative analysis of 13 angles and 167 distances of the child’s skull allowed for the determination of the growth pattern of the skull in such children. The CI was significantly lower in children with scaphocephaly and trigonocephaly. In comparison to healthy children, a reduction of the measured dimensions in upper part of facial skeleton in children with craniosynostosis has been observed. Following dimensions were decreased: nasal orbital angle, which fluctuated in the range of 108°±6° in healthy children–when measured in children with craniosynostosis it was significantly lower and amounted to 99°±5° (the largest difference of dimensions has been observed in children of 6–8 months of age and the lowest one in children of 3–6 months of age), angle of frontal bone (*nasion-fno*) on the right and left–the biggest differences occur in dimensions in children 3–6 months of age. The measured index of external width of the eye sockets and the skull width as well as interorbital angle (angle between *nasion-mf–*left and right) was significantly lower in children with trigonocephaly, in comparison to healthy children. In our study, we chose a different set of measured parameters than Kowalewska. BCAW, LPBW, RPBW as well as ABW are important for an evaluation of any upper airways stenosis, which may accompany craniosynostosis. In our study, a decrease of the dimension of the nasal cavity in children with nonsyndromic craniosynostosis, which is a part of upper massif of facial skeleton, has been confirmed as well. The size of the nasal cavity is subject to personal differences, however, changes in size are more often due to pathological causes. The study conducted by Djupesland et al. [29] demonstrated that the size of the air space in the nasal cavity did not depend upon the sex, but had positive statistically significant correlation with child’s head size [29]. Our research confirmed that for both children with isolated craniosynostosis and healthy children no statistically significant differences between the sexes occur, with exceptions of the dimensions BCAW and RPBW in children under 6 months of age and LPBW in children between 3 and 6 months of age. The absence of differences in the analyzed facial skeleton dimensions in children was also confirmed by other researchers [26]. To set the standards of plagiocephaly and brachycephaly diagnosis, Wilbrandt et al. [20] evaluated cranial index on 3D reconstructions in a group of healthy children under 12 months and in children with nonsyndromic craniosynostosis. The results also showed no differences between the sexes in skull dimension in children with craniosynostosis.

In patients with syndromic craniosynostosis, reductions of the diameters of nasal cavity are generally considered to be the major cause of upper airway obstruction. Almost 50% of children with Apert, Crouzon, or Pfeiffer syndrome develop obstructive sleep apnea (OSA) [13]. It is still unknown if similar disorders can be found in children suffering from isolated craniosynostosis. Congenital bony nasal stenosis (CBNS) is a very rare but life-threatening cause of airway obstruction in neonates and infants. CBNS can happen as an isolated anomaly or as part of an associated condition like craniofacial anomalies. Liew et al. presented 4 cases of craniosynostosis with bilateral nasal cavity stenosis, what may suggest that children with the abovementioned deformations can also present abnormalities concerning the nasal cavity [30].

A rate of symptomatic restenosis after extended strip craniectomy (ESC) and radiologic changes were estimated in a study of Bonfield et al. In his retrospective study comprising 238 infants with nonsyndromic sagittal synostosis two morphometric parameters, cranial index (CI) and nasofrontal angle (NFA), were compared before and after a surgical correction. The surgical treatment of ESC resulted in a mean increase of CI from 0.68 to 0.75 accompanied by a mean increase of NFA from 127 to 133.The treatment procedure performed in those infants required neither direct frontal bone resection, nor frontal orbital osteotomy. A significant increase of CI was achieved without an adjunctive helmet treatment [17]. Water et al. [19] measured the upper airway volume using Dolphin 3D Software and found a significant increase in airway volume in patients with craniosynostosis after Le Fort III osteotomy. The airway was divided into the oropharynx (compartment A) and the nasal passage (compartment B). The average airway volume before the procedure was 30.054 mm (for compartment A—10.090 mm and B—19.963 mm). The average airway volume after the procedure was 36.574 mm (for compartment A—10.040 mm and B—27.351 mm). Patients with syndromic craniosynostosis often suffer from severe underdevelopment of the midface due to premature fusion of the calvarial sutures.

The aim of our study was to complete the knowledge about morphometric dimensions of the nasal cavity in children with craniosynostosis. The possible clinical significance of the abnormal dimensions of the posterior nasal cavity in these children still requires further research. However, it is known that infants breathe only through the nose. Incorrect development of the nasal cavity may contribute to the more frequent infections, impede therapies in intensive care units and otolaryngology units, or even be the cause of respiratory failure. The development of the skull is inseparably associated with the development of the brain. Djupesland pays attention to the importance of the complicated anatomy of the nasal cavity during using the nose as the route of drug delivery to the brain bypassing the blood/ brain barrier. The results of our research complement the works of this author. Possible anatomical limitations in the dimensions of the nasal cavity may affect the transport of medications. This aspect also requires farther research [31].

The exposure to ionizing radiation during CT imagining, when carrying out studies in the area of morphometry also requires explanation. We conducted only retrospective study. The research group consists of children referred to the CT for clinical indications by a neurosurgeon, who qualified them for cranial plastic surgery. CT scanning was performed with a thin layer, because the main goal was to obtain high quality 3D reconstruction. Reconstructions were used to predict the shape of the skull after surgery, using a special computer program. Assessment of the course of dural venous sinuses was the second aim of imagining, because they can be a source of complications during the surgical operation [32]. For our publication, no additional tests were performed—it is retrospective research and we did not expose children to the additional CT scans. To reduce the dose, a pediatric protocol was used and the phase before radiocontrast application was abandoned. Currently, it is possible to assess the shape of the skull in MR sequences, without exposure to radiation, which we use in our hospital. However, the previously obtained material was used in our study to carry out additional measurements.

Deviations in anthropometric indicators of the skull may reflect structural anomalies in both forming of the calvarium as well as the deformations of facial skeleton and its upper part massif of the facial skeleton. For these reasons, many researches support the monitoring of anthropometric parameters and calculating cranial indices for patients with deformations of facial skeleton. To sum up, the reduction of nasal cavity dimensions in children with nonsyndromic craniosynostosis may be associated with the premature atresia of cranial sutures, which disrupts growth of nasal cavity in children under 3 years old.

## Conclusions

Our study shows that in children with nonsyndromic craniosynostosis the diameter of pyriform aperture and bony choanal aperture were lower than in healthy children. It may indicate a fronto-orbital anomaly in these children. Anthropometric measurements of the skull based on CT scans are an unbiased way to assess restrictions and other deformations of skull in children and have significant clinical value. The presented here data may contribute to a better understanding of the skull growth processes in children with nonsyndromic craniosynostosis.

## Supporting information

S1 Table A list of patients with craniosynostosis, presenting types of craniosynostosis and results of measurements. (XLS)
